# DISCRIMINATION IN OLDER ADULTHOOD: UNDERSTANDING THE IMPACT ON PURPOSE IN LIFE

**DOI:** 10.1093/geroni/igad104.1663

**Published:** 2023-12-21

**Authors:** Megan Wilson, Isaiah Spears, Sara Norton, Ryan Bogdan, Thomas Oltmanns

**Affiliations:** Washington University in St. Louis, St. Louis, Missouri, United States; Washington University in St. Louis, St. Louis, Missouri, United States; Washington University in St. Louis, St. Louis, Missouri, United States; Washington University in St. Louis, St. Louis, Missouri, United States; Washington University in St. Louis, St. Louis, Missouri, United States

## Abstract

Experiences of discrimination negatively impact individuals across a variety of outcomes, and may similarly impact individuals’ sense of purpose, meaning the feeling that one has meaningful goals and direction in life (Ryff et al., 2003). However, counter to a deficit perspective on marginalization, research also suggests that discrimination may be less negatively associated with sense of purpose for minority groups (Wilson & Hill, 2023a). One possible reason is that members of minority groups may be more likely to find purpose through discrimination (Wilson & Hill, 2023b). Thus, the current study examined a sample of 1630 older adults (Mean Age = 73.08) in order to understand the relationship between discrimination and sense of purpose, and moderators of this association. Results indicate that discrimination is negatively associated with sense of purpose. Contradicting past work, this effect did not differ for racial minority group members. However, we do find that individuals with higher education show a less negative association between discrimination and sense of purpose, suggesting education as a potential pathway to overcome discrimination in the pursuit of meaningful life goals. Moreover, while discrimination was negatively associated with health, sense of purpose moderated this relationship, suggesting a potential for sense of purpose to serve as a resilience factor to protect individuals from harmful effects of discrimination. Findings will be discussed in terms of how we may help older adults foster a sense of purpose and life direction, and in terms of the broader implications on older adults’ well-being and health.

